# A case of bronchial asthma as an immune-related adverse event of pembrolizumab treatment for bladder cancer: A case report

**DOI:** 10.1097/MD.0000000000028339

**Published:** 2022-01-14

**Authors:** Kazuyuki Hamada, Kiyoshi Yoshimura, Kazuhiko Oshinomi, Yuya Hirasawa, Hirotsugu Ariizumi, Ryotaro Ohkuma, Midori Shida, Yutaro Kubota, Hiroto Matsui, Tomoyuki Ishiguro, Takehiko Sambe, Hiroo Ishida, Atsushi Horiike, Satoshi Wada, Sanju Iwamoto, Naoki Uchida, Yoshio Ogawa, Shinichi Kobayashi, Takuya Tsunoda

**Affiliations:** a Department of Medicine, Division of Medical Oncology, Showa University School of Medicine, Tokyo, Japan; b Department of Clinical Immuno Oncology, Clinical Research Institute for Clinical Pharmacology and Therapeutics, Showa University, Tokyo, Japan; c Department of Urology, Showa University School of Medicine, Tokyo, Japan; d Department of Pharmacology, Showa University School of Medicine, Tokyo, Japan; e Department of Clinical Diagnostic Oncology, Clinical Research Institute for Clinical Pharmacology and Therapeutics, Showa University, Tokyo, Japan; f Division of Physiology and Pathology, Department of Pharmacology, Toxicology and Therapeutics, Showa University School of Pharmacy, Tokyo, Japan; g Clinical Research Institute for Clinical Pharmacology and Therapeutics, Showa University, Tokyo, Japan.

**Keywords:** anti-PD-1 antibody, asthma, bladder cancer, immune-related adverse events, pembrolizumab

## Abstract

**Rationale:**

Bladder cancer is one of the most common cancers worldwide. The anti-programmed cell death protein 1 (PD-1) antibody pembrolizumab, which is an immune checkpoint inhibitor (ICI), has improved survival in bladder cancer. We report a case of bladder cancer that had a high antitumor effect with anti-programmed cell death PD-1 antibody pembrolizumab, an ICI, but asthma occurred an immune-related adverse event (irAE).

**Patient concerns:**

A 70-year-old female patient was diagnosed as unresectable bladder cancer who was indicated for ICI treatment.

**Diagnosis:**

After ICI administration as a treatment for bladder cancer, the patient had a grade 3 asthma attack. Cytotoxic T lymphocyte antigen 4 (CTLA-4) in CD4^+^ FOX3^+^ T cells was upregulated in the early phase before the development of asthma attacks. Moreover, T-cell immunoglobulin and mucin domain 3 (TIM-3) was upregulated in all memory T cells among CD4^+^ T cells. However, no change in the expression of TIM-3 was observed in any CD8^+^ T-cell subtype. In contrast, the proportion of CD161^-^ T helper 17 cell (Th17) cells increased.

**Interventions:**

The patient was treated with betamethasone, montelukast, salbutamol nebulization, and a combination of salmeterol (50 μg) and fluticasone (500 μg) (SFC).

**Outcomes:**

The patient's wheezing resolved, and her peak flow rate reached 100% of the predicted value; therefore, the patient continued treatment with SFC and montelukast and was discharged from the hospital.

**Conclusion:**

Increases in CTLA-4 and TIM-3 expression in CD4^+^ T cells (not CD8^+^), as well as an increase in Th17 cells, may reflect asthma-related inflammation activity. Immune-related adverse events during immune checkpoint inhibitor administration may be predictive markers of antitumor efficacy.

## 1 Introduction

The expression of programmed death-ligand 1 in cancer cells sends inhibitory signals to T cells through programmed cell death protein 1 (PD-1), allowing tumor cells to evade the immune system.^[1]^ The anti-PD-1 antibody pembrolizumab activates tumor-specific CD8^+^ T cells, and has been approved for the treatment of patients with advanced urothelial carcinoma. However, immune-related adverse events (irAEs) are caused by immune checkpoint inhibitors (ICIs), such as anti-PD-1 antibodies. Typical irAEs include endocrine disorders, rash, diarrhea, liver dysfunction, and interstitial pneumonia.^[2]^ Asthma is also an extremely rare irAE.^[3]^

We encountered a patient who experienced asthma associated with pembrolizumab and achieved marked antitumor efficacy. To the best of our knowledge, this is the first report of asthma occurring as an irAE of pembrolizumab in invasive bladder cancer, and we report the expression of immune checkpoints (ICs) in peripheral blood memory T cells before and after asthma onset.

## 2 Case presentation

The patient was a 70-year-old woman with invasive bladder cancer and diabetes mellites. She had a history of smoking ten cigarettes per day for 40 years, with no history of allergies, including asthma. However, her father and daughter had asthma. Treatment with carboplatin plus gemcitabine was followed by pembrolizumab (200 mg/body once every 3 weeks) due to primary tumor regrowth.

Abdominal computed tomography (CT) after the second cycle of pembrolizumab treatment showed a reduction in the primary tumor and metastases.

**Figure 1:**
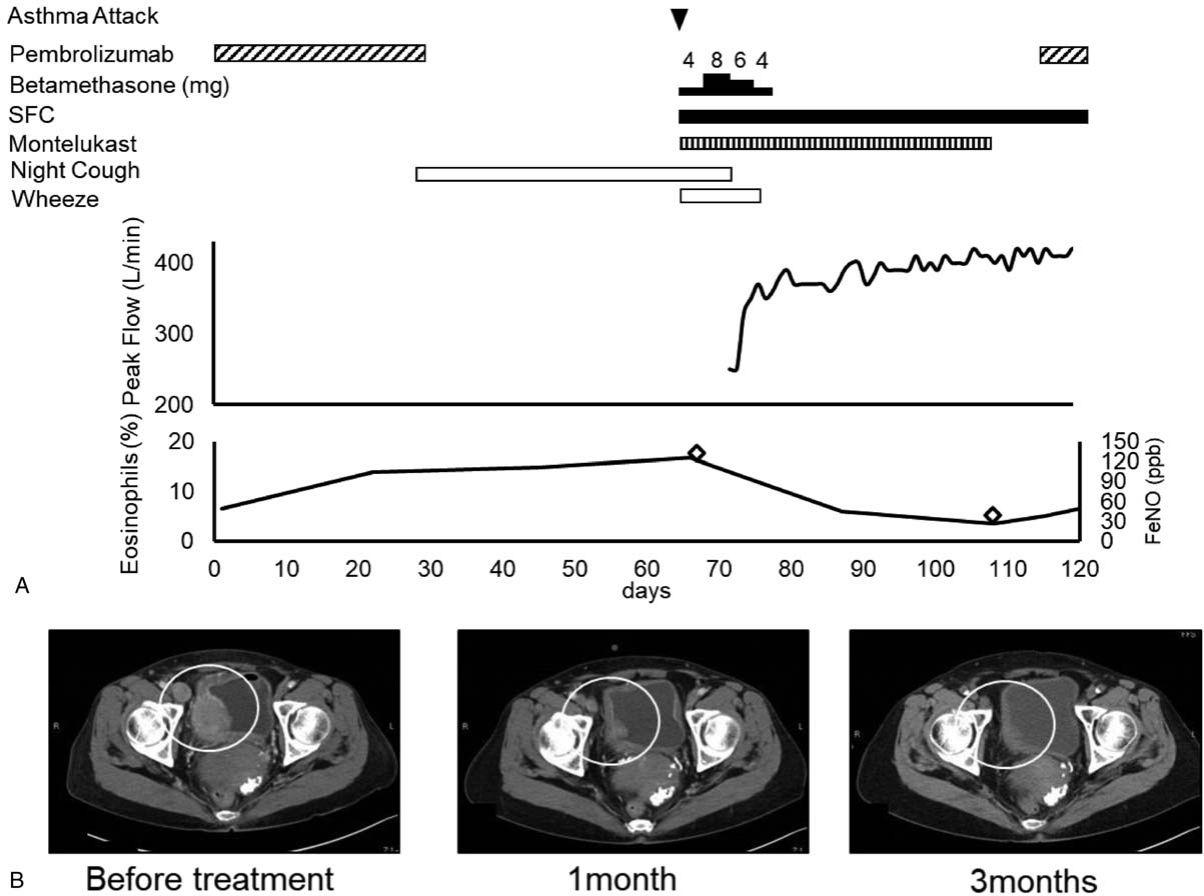
Clinical course. (A) Treatment of asthma and bladder cancer. (B) Efficacy of bladder cancer treatment. Computed tomography of primary bladder cancer and lymph node metastasis. Arrowhead, asthma attack; FeNO, rhombus; circle, bladder cancer.

The patient had started experiencing coughs at night after day 9 of the second treatment cycle (Fig. 1A, B). Wheezing was observed when the patient was admitted to the fourth cycle of pembrolizumab treatment. Her peripheral oxygen saturation in room air was 95%. Chest radiography revealed no abnormalities, and CT showed several small nodules of lung metastases and mild emphysematous changes. Blood tests revealed a white blood cell count of 15000/μL, a neutrophil count of 6700/μL, a lymphocyte count of 2120/μL, and an eosinophil count of 1920/μL (16.7%). The serum total IgE level increased to 291 IU/mL. C-reactive protein levels were normal. The myeloperoxidase-antineutrophil cytoplasmic antibody test result was negative. Hence, no findings were indicative of the infection. The patient's vital capacity was 2.89 L, forced expiratory volume in 1 second was 1.51 L, and the forced expiratory volume in 1 second was 52.25%. However, the fraction of exhaled nitric oxide (FeNO), which is indicative of eosinophilic airway inflammation, was as high as 131 ppb (reference value, 36.8 ppb). Nevertheless, echocardiographic findings were normal. The patient was diagnosed with asthma onset and was admitted to the hospital for an asthma attack. She was treated with betamethasone, montelukast, salbutamol nebulization, and a combination of salmeterol (50 μg) and fluticasone (500 μg) (SFC). Her wheezing resolved, and her peak flow rate reached 100% of the predicted value; therefore, the patient continued treatment with SFC and montelukast and was discharged from the hospital.

Forty-nine days after the asthma attack, the patient's FeNO levels decreased to 38 ppb, and she no longer had asthma symptoms. Hence, pembrolizumab was reintroduced, and the patient's asthma remained stable with SFC treatment even after pembrolizumab administration.

We investigated the relationship between asthma and the expression of immune checkpoints in peripheral blood T-cell subsets, which were measured by flow cytometry.

**Figure 2:**
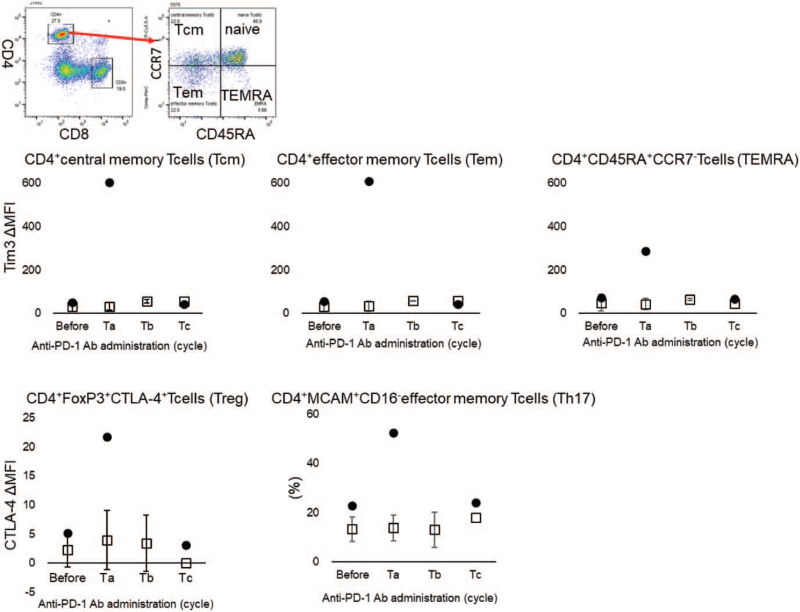
Immune checkpoints on CD4+ memory T cells and the proportion of CD161^-^ Th17 effector memory T cells. • In the present case, □ in control cases. The results represent the mean ± SD. The horizontal axis shows the number of cycles of anti-PD-1 antibodies in the figure. Before the first cycle of anti-PD-1 antibodies administration, Ta; before the second cycle, Tb; before third cycle or fourth cycle, Tc; before the fifth or sixth cycle.

The present case was compared with 3 other cases in which the patients were treated with anti-PD-1 antibodies that had no effect and no irAEs (Fig. 2, Table 1). The expression of T cell immune checkpoints during the development of asthma as an irAE is not known. Generally, the expression of immune checkpoints increases with T cell activation. Since this patient developed asthma triggered by the administration of PD-1 antibody, the expression of immune checkpoint on T cells was expected to be elevated after the administration of PD-1 antibody. Patients who did not develop asthma and did not respond to PD-1 antibodies were selected as controls (Table 1 control1, 2, 3). Interestingly, the expression of T-cell immunoglobulin and mucin domain 3 (TIM-3), a typical immune checkpoint, was upregulated in CD4 T cells after PD-1 antibody treatment in the present patient compared to controls. In contrast, no increase in TIM3 was observed in the control group, but no change in the expression was observed in any CD8+ T cell subtype.

**Table 1:** Patient characteristics the best overall response was assessed using the response evaluation criteria in solid tumors, version1.1. irAE = immune-related adverse events.

Case	Age	Gender	Type of malignancy	Histology	Clinical Stage	Treatment	Best Overall Response	irAE
This case	70	Female	Bladder cancer	Urothelial cancer	IV	Pembrolizumab	Partial Response	Asthma
Control 1	66	Male	Gastric cancer	Adenocarcinoma	IV	Nivolumab	Progressive Disease	None
Control 2	60	Male	Esophagogastric junction cancer	Adenocarcinoma	IV	Nivolumab	Progressive Disease	None
Control 3	72	Male	Lung cancer	Adenocarcinoma	IV	Pembrolizumab	Progressive Disease	None

It was unique to the present case that cytotoxic T lymphocyte antigen 4 (CTLA-4) in CD4^+^ Forkhead box P3 (Foxp3) ^+^ T cells were upregulated in the early phase before the development of asthma attacks. Tregs are thought to be activated to suppress inflammation in asthma. These results suggest that the PD-1 antibody activates CD4+ T cells and is involved in the development of asthma. Focusing on the activation of CD4 T cells, we investigated the proportion of activated T helper 17 cell (Th17) cells, which are a subtype of CD4+ T cells associated with allergic inflammation. Th17 cells were elevated only in the asthma cases. The activation of Th17 was involved in the development of asthma as an irAE in the present case.

## 3 Methods

### 3.1 Flow cytometric analysis

Flow cytometric analysis was performed to confirm the expression of each factor. After washing, the cells were suspended in PBS with FBS (2%) at a concentration of 2 × 105 cells per 100 μL.

For staining, the following primary antibodies were used: CD45RA, mouse IgG2b, κ, and FITC. CD274 programmed death-ligand 1, mouse IgG1, κ, and PE-Cy7. CD366 (Tim-3) mouse IgG1, κ, APC CD4 mouse IgG1, κ, and APC-Cy7. CD8a mouse IgG1, κ, BV510 CD152 (CTLA-4) mouse IgG2a, κ, and BV421. CD4 Mouse IgG1, κ, PerCP-Cy5.5. Foxp3 mouse IgG1, κ, and AF647. ICOS Armenian Hamster IgG, PE-Cy7 (BioLegend, CA, USA), CD197 (CCR7), mouse IgG2a, and PerCP-Cy5.5. CD3 mouse IgG2a, κ, BV510 (BD Biosciences, CA, USA)

The samples were analyzed with BD FACSVerse (BD Biosciences), and the data were analyzed using FlowJo software (TreeStar, OR, USA).

The relative fluorescence intensity (RFI) was calculated as follows: RFI = ([mean fluorescence intensity [MFI] of the molecule] – MFI of the corresponding isotype control)/(MFI of corresponding isotype control).

## 4 Study approval

This study was approved by the Declaration of Helsinki and the ethical review board of the School of Medicine, Showa University (no. 2253). Sampling was performed during routine clinical procedures. Patients provided written informed consent for participation in the study.

## 5 Discussion

In the present case, asthma developed as a rare irAE of an anti-PD-1 antibody. Previously, Maeno et al reported a case of nivolumab-induced asthma in a patient with non-small cell lung cancer (NSCLC).^[3]^ The case presented with eosinophilic inflammation, and the elevation in the number of peripheral blood eosinophils, serum total Immunoglobulin E, and the FeNO was similar to our case. Treatment with inhaled corticosteroids and long-acting beta-2 agonists improved the symptoms of asthma.^[3]^ On the other hand, Ogawa et al reported pembrolizumab-induced fatal CD8^+^ infiltrated asthma-like bronchitis in a patient with NSCLC.^[4]^ Mitropoulou et al reported 2 cases of severe bronchitis attributable to nivolumab with or without ipilimumab in patients with melanoma and NSCLC.^[5]^ Airway diseases such as asthma and bronchitis have been recognized as a new pattern of irAE; hence, asthma-like symptoms should be monitored when an anti-PD-1 antibody is administered.

In the present case, irAEs and clinical effects were observed simultaneously. Progression-free survival (PFS) continued for more than 6 months and the results achieved were even better than those seen in the KEYNOTE 045 study of urothelial cancer, which reported a median overall survival of 10.3 months, a median PFS of 2.1 months, and an overall response rate of 21.1%.^[2]^ The patient experienced a strong therapeutic effect against the target cancer, along with a grade 3 asthma attack. Similarly, there are some reports that patients with irAEs from anti-PD-1 antibody treatment experience a greater therapeutic effect than patients without irAEs.^[6]^

Moreover, this represents the first case in which the expression of ICs in peripheral blood memory T cells was described before and after pembrolizumab irAE. CTLA-4 is expressed upon the activation of T lymphocytes, binds to both CD80 and CD86 ligands, and is expressed on antigen-presenting cells to suppress activated T cells.^[7]^ Among regulatory T (Treg) cells, CTLA-4 showed a transient increase in expression immediately after the initiation of anti-PD-1 antibody treatment. This finding suggests that Tregs are activated to suppress asthma inflammation. TIM-3 is widely expressed not only in type I helper T cells but also in Th17 cells, natural killer cells, NK T cells, dendritic cells, and macrophages.^[7]^ In our case, the upregulation of TIM-3 on CD4^+^ memory T cells implies the activation of CD4^+^ memory T cells.

Furthermore, Th17 cells increase in allergic asthma patients,^[8]^ and it should be noted that Th17 elevation was observed in our case. Th17 cells may also be involved in the pathogenesis of irAE-associated asthma and allergic asthma.

In terms of tumor immunity, focusing on memory T cells has a significant impact on the tumor microenvironment. In addition, CD4^+^ T cell reactions in asthma should be considered as irAEs.

Here, we report a case of asthma as an irAE of pembrolizumab treatment. The patient had eosinophilia, a family history of asthma, and elevated IgE levels. Atopic predisposition may be a risk factor for irAEs in asthma. Increases in CTLA-4 and TIM-3 expression not on CD8^+^ but CD4^+^ T cells, as well as an increase in Th17 cells, may reflect asthma-related inflammation activity. Nevertheless, this was a single case report, and the accumulation of further data is still necessary.

## Author contributions

**Conceptualization:** Kiyoshi Yoshimura.

**Data curation:** Kazuhiko Oshinomi, Ryotaro Ohkuma, Yutaro Kubota, Tomoyuki Ishiguro, Hiroo Ishida, Atsushi Horiike, Satoshi Wada.

**Funding acquisition:** Kiyoshi Yoshimura.

**Investigation:** Yuya Hirasawa, Midori Shida, Takehiko Sambe.

**Supervision:** Sanju Iwamoto, Yoshio Ogawa, Shinichi Kobayashi, Takuya Tsunoda.

**Validation:** Hirotsugu Ariizumi, Hiroto Matsui, Takehiko Sambe, Naoki Uchida.

**Writing – original draft:** Kazuyuki Hamada.

**Writing – review & editing:** Kiyoshi Yoshimura.
